# The N-Terminal Domain of Y-Box Binding Protein-1 Induces Cell Cycle Arrest in G2/M Phase by Binding to Cyclin D1

**DOI:** 10.1155/2009/243532

**Published:** 2010-04-14

**Authors:** Payal Khandelwal, Mythili K. Padala, John Cox, Ramareddy V. Guntaka

**Affiliations:** Department of Molecular Sciences, The University of Tennessee Health Science Center, Memphis, TN 38163, USA

## Abstract

Y-box binding protein YB-1 is a multifunctional protein involved in cell proliferation, regulation of transcription and translation. Our previous study indicated that disruption of one allele of Chk-YB-1b gene in DT-40 cells resulted in major defects in the cell cycle. The abnormalities seen in heterozygous mutants could be attributed to a dominant negative effect exerted by the disrupted YB-1 allele product. To test this hypothesis the N-terminal sequence of the YB-1 was fused with the third helix of antennapedia and the green fluorescent protein. These purified fusion proteins were introduced into rat hepatoma cells and their effect on cell proliferation was studied. Results indicate that the N-terminal 77 amino acid domain of the YB-1 protein induced the cells to arrest in G2/M phase of the cell cycle and undergo apoptosis. Additional deletion analysis indicated that as few as 26 amino acids of the N-terminus of YB-1 can cause these phenotypic changes. We further demonstrated that this N-terminal 77 amino acid domain of YB-1 sequesters cyclin D1 in the cytoplasm of cells at G2/M phase of cell cycle. We conclude that the N-terminal domain of YB-1 plays a major role in cell cycle progression through G2/M phase of cell cycle.

## 1. Introduction

Y-box binding proteins are members of the Cold Shock Domain (CSD) super family of proteins [1]. They are involved in the regulation of transcription [2, 3], modulation of translation [4], DNA repair [5], and drug resistance [6–8], stress response to extracellular signals [9, 10] and in an early stage of embryogenesis [11, 12]. Several studies also showed up-regulation of YB-1 protein levels in proliferating cells in comparison to quiescent or non-proliferating cells [1, 13]. YB-1 activates many genes implicated in cell proliferation including DNA polymerase *α* [14], proliferating cell nuclear antigen (PCNA) [15, 16], thymidine kinase and topoisomerase II [15, 17]. However, the mechanism by which YB-1 promotes cell proliferation is not understood. 

 Knock-out studies have been done to gain insight into the function of YB-1 in cell proliferation. We showed previously that targeted disruption at the 5′ end of one allele of chicken YB-1 gene in DT-40 cells resulted in major cell cycle defects, including a slower doubling time, increased genomic DNA content, increased cell size and apoptosis in a fraction of the cell population [18]. In contrast, another group found that YB-1^+/−^ heterozygous mutants did not show any apparent growth defects, whereas YB-1^−/−^ cells exhibited a markedly reduced growth phenotype [19]. Targeted disruption at the 3′ end of one allele of YB-1 rendered mouse embryonic stem cells more sensitive to cisplatin and mitomycin C without any apparent growth defects [20]. Furthermore, down regulation of YB-1 by shRNA resulted in a significant reduction in the rate of proliferation and increased rate of apoptosis [21]. These studies indicated that the amino terminus of YB-1 may be involved in cell proliferation. A definitive role for YB-1 in cell proliferation has been demonstrated by knocking out both alleles of YB-1 in mice [12]. These mice are embryonic lethal, indicating a non-redundant role for YB-1 in early embryonic development. Further studies with YB1^−/−^ fibroblasts showed greatly reduced cell proliferation and altered cell morphology, demonstrating a critical role for YB-1 in cell proliferation [12].

 In our earlier studies [18] we speculated that the altered phenotypes we observed in DT-40 cells might be due to a dominant negative effect exerted by a putative truncated protein encoded by the disrupted YB-1 allele. If this assumption is correct, then introduction of the N-terminal domain of YB-1 into cells should mimic the phenotypic changes observed in the mutant DT40 cells [18]. Therefore, we constructed clones expressing either the 26 or 77 amino acid polypeptide sequence corresponding to the N-terminus of YB-1. We also made an internal deletion which removed the alanine and proline-rich sequence of the N-terminal 77 amino acids of YB-1. These polypeptides were fused with the antennapedia homeodomain, which facilitates receptor independent uptake of the proteins into cells and a reporter green fluorescent protein (GFP) to monitor the uptake and cellular localization of the proteins. These fusion proteins were introduced into rat hepatoma cells and their effect on cell growth studied. Our results clearly indicated a role for YB-1 in cell proliferation which is mediated by the N-terminal domain of YB-1 probably by sequestering cyclin D1 in the cytoplasm, thus blocking cell cycle progression from G2/M.

## 2. Materials and Methods

**Antibodies and Reagents**

All the chemical reagents, unless otherwise specified were from Sigma-Aldrich (St. Louis, MO). Cell culture media were from Invitrogen-GIBCO (Carlsbad, CA). The YB-1 polyclonal antibody was generated for our lab by Sigma. Primary antibodies and fluorescent-labeled secondary antibodies were obtained from Invitrogen-Molecular probes (Carlsbad, CA).

**Cloning and Expression of APGFP-Fusion Proteins**

The pUC9 vector was modified in our lab and the three sequences of YB-1 were cloned in frame with an upstream sequence coding for the 16 amino acid long antennapedia homeodomain (AP) and downstream sequence coding for GFP (Figure 2(a)).The three clones APYB77GFP, APYB36GFP, APYB26GFP and the control clone APGFP, which lacked the YB-1 sequence, were transformed into *E. coli* BL21DE3 cells. Log phase cultures were induced overnight with 1 mM IPTG and proteins were purified.

**Purification of Proteins**

Inclusion bodies were isolated and solubilized as described [22] and the fusion proteins were further purified from the supernatants using DEAE Sephacel ion-exchange chromatography at 4°C as described [23]. The expected fusion proteins were detected in fractions eluted with 0.3 M NaCl. These fractions were dialyzed overnight with 4 changes of TNG buffer (50 mM Tris-HCl, pH 8.0, 100 mM NaCl and 5% glycerol). Purity of the proteins was determined by running triplicates of the protein fractions on a 15% SDS-PAGE gel followed by coomassie staining or silver staining and western blotting with GFP antibody. Protein concentration was determined using BCA protein assay kit (Pierce- Rockford, IL).

**Synchronization and Treatment of Cells**

Rat Hepatoma cells (ATCC-H-411E) were grown in Minimum Essential Medium supplemented with 10% FBS, 100 U/mL Penicillin and 100 *μ*g/mL Streptomycin, in a humidified incubator maintained at 37°C and 5%  CO_2_. Cells fed every day were subcultured 1 : 5 on confluency, using 0.05% trypsin-EDTA. For experimental analysis, cells were incubated with 40 *μ*g/mL of the purified proteins for the indicated time periods. Double thymidine block was used for synchronization of cells in G1 as described [24]. For G2/M phase synchronization cells were incubated with 1 to 2.5 *μ*g/mL nocodazole for 14 to 24 hrs.

**Immunoblotting**

Cells were washed and lysed with RIPA buffer following the published protocol [25]. For nuclear and cytoplasmic fractions, the cells were scraped in 100 *μ*L ice cold PBS and centrifuged at 500 g for 5 min. The cell pellet was resuspended in 100 *μ*L Buffer A (50 mM NaCl, 10 mM HEPES pH-8.0, 500 mM sucrose, 1 mM EDTA, 0.2% Triton-X-100, freshly added protease inhibitors and 7 mM *β*-mercaptoethanol), vortexed at high speed for 45 sec and centrifuged at 2000 g for 2 min at 4°C. The resulting supernatant was used as cytoplasmic extract. The pellet was resuspended in 100 *μ*L Buffer B (Buffer A with 25% glycerol and 0.1 mM EDTA) and nuclei were pelleted at 2000 g for 2 min at 4°C. The pellet was dissolved in 50 *μ*L Buffer C (Buffer B with 350 mM NaCl), incubated on ice for 30 min with intermittent high speed vortexing and spun down at 11000 g for 15 min at 4°C. The supernatant was diluted to 100 *μ*L with PBS and used as nuclear extract. Equal amounts of samples were analyzed on SDS-PAGE gel. 1 : 7500 dilution of Antirabbit GFP antibody was used as primary antibody for overnight incubation at 4°C. 1 : 15000 dilution of HRP-conjugated Antirabbit IgG was used as secondary antibody for 2 hr at room temperature. The blot was developed using chemiluminescence kit (Pierce, Rockford, IL) according to the manufacturer's protocol.

**Immunoprecipitation**

Equal amounts of whole cell lysates or nuclear and cytoplasmic extracts were diluted to 500 *μ*L with lysis buffer and incubated with 1 : 2000 dilution of cyclin A, B1, D1 D2 and GFP antibodies overnight at 4°C. Equal amount of Protein A Sepharose beads (Amersham Biosciences—Piscataway, NJ) was added to each sample and incubated for 2 hr at room temperature. The beads were pelleted down at 8000 g for 4 min and the pellet was washed with lysis buffer twice. The resulting pellet was resuspended in sample buffer and loaded onto SDS-PAGE gels.

**Immunocytochemistry and Indirect Immunofluorescence**

Cells were rinsed twice with Phosphate buffered saline (PBS), fixed for 15 min. with 4% *p*-formaldehyde, washed twice with PBS and stored at 4°C. Cells were blocked with 5% goat serum and 3% BSA in PBS for 30 min. and rinsed with PBS thrice. Then the cells were incubated with primary antibodies Antirabbit GFP (1 : 500) or Antirabbit YB-1 (1 : 300) and Antimouse cyclin D1 (1 : 300) for 3 hrs followed by several washes with PBS. Subsequently cells were incubated with secondary antibodies FITC conjugated Antirabbit IgG (1 : 500) and Texas-Red conjugated Antimouse IgG (1 : 500) for 1 hr at room temperature in dark room. Coverslips were washed several times with PBS and once with water to remove any salts and left to air-dry for 45 min in absence of light. The coverslips were mounted onto glass slides using Vectashield Mounting Medium containing DAPI (Vector Laboratories—Burlingame, CA) to counterstain nuclei. The slides were stored at −20°C until analyzed by microscopy.

**Confocal Microscopy**

Confocal images were captured on LSM 510 laser-scanning three-color microscope (Carl Zeiss, Jena, Germany) using Argon laser at 75% output and HeNe1 laser at excitation wavelengths 488 nm and 543 nm, respectively. Optical slice thickness was set to 0.3 *μ*m, magnification to 40× and sections presented were taken approximately at the mid height level of cells. Photomultiplier gain and laser power were identical within each experiment. Apoptotic blebs were seen using Axioplan 2 epiflorescent microscope (Zeiss, Germany). DAPI fluorescence was captured using the 63× magnification lens of the microscope. Axiovision software was used to capture zvi-stacks of images. Exposure was set using control nuclei and was fixed for each experiment. A minimum of 100 stained cells were scored for each experiment. All images were analyzed and adjusted for contrast in Adobe Photoshop 5.0 (San Jose, CA).

**DNA Fragmentation**

Exponentially growing cells were incubated with the fusion proteins for 24, 48 and 72 hr. Analysis of DNA fragmentation was done as described [18].

**FACS Analysis**

Cells were harvested by trypsinization or scraping on ice and pelleted at 500 g for 5 min. They were washed with PBS, fixed with ice cold 70% EtOH, incubated on ice for 45 min and centrifuged at 500 g for 5 min. The pellet was washed with Hanks Balanced Salt Solution (HBSS) containing 1% BSA, resuspended in propidium iodide buffer (1 : 1 dilution of 20 ug/mL of PI in HBSS and 1 mg/ml RNAse A) and incubated for 30 min in a 37°C water bath. Samples were analyzed using a Becton Dickinson FACS Calibur. For analysis of apoptotic cells, Aposcreen Annexin V-FITC kit (Southern Biotechnology Associates Inc., Birmingham, AL) was used according to the manufacturer's protocol. Cell debris was excluded from the FACS analysis by appropriate forward and light scatter gating. Cell cycle profiles were analyzed and bar graphs were plotted using Microsoft Excel.

## 3. Results

### 3.1. APYBGFP and APGFP Fusion Proteins Are Internalized and Stable Inside Cells

**Figure 1:**
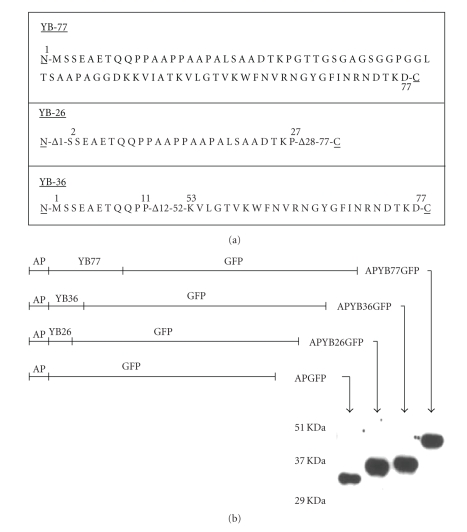
Schematic representation of the fusion constructs and the amino acid sequence of truncated YB-1 proteins. (a) Amino acid sequence of the N-terminal region of each of the truncated YB-1 protein. Note an internal deletion in APYB36GFP which removes the alanine-proline sequence (b) Line drawing of each fusion containing antennapedia, YB-1 and GFP and size of E. coli expressed fusion proteins as analyzed by PAGE followed by western blot analysis.

Earlier we disrupted one allele of the YB-1 gene in DT-40 cells and observed profound cell cycle defects [18]. We proposed that this effect could be due to truncated proline-rich polypeptide encoded within exon 1 and produced by the disrupted allele. In order to test whether the putative truncated protein could be responsible for the previously observed results, we developed an expression cassette containing the amino-terminal 77 amino acids of YB-1 inserted between the 16 amino acid antennapedia peptide and a GFP reporter. The amino acid sequence of these clones is shown in Figure 1(a). This construct will be referred as APYB77GFP (Figure 1(a) Line drawing). Two other fusions containing the 26 N-terminal amino acids of YB-1 (APYB26GFP) and a clone with an internal deletion that removed the alanine-proline-rich sequence from the N-terminal 77 amino acids of YB-1 (APYB36GFP) were also generated (Figure 1(b) Line drawings). These fusion proteins were purified to more than 80% homogeneity as assessed by polyacrylamide gel electrophoresis and their sizes were confirmed by western blotting (Figure 1(b)). 

**Figure 2:**
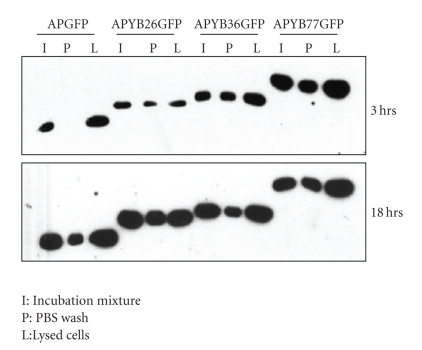
Evidence that APYBGFP and APGFP fusion proteins get internalized into cells. Whole cell extracts of rat hepatoma cells incubated with each fusion protein for 3 and 18 hr were analyzed by western blotting using anti-GFP antibody. Details of the amounts used are discussed in Section 2.

**Figure 3:**
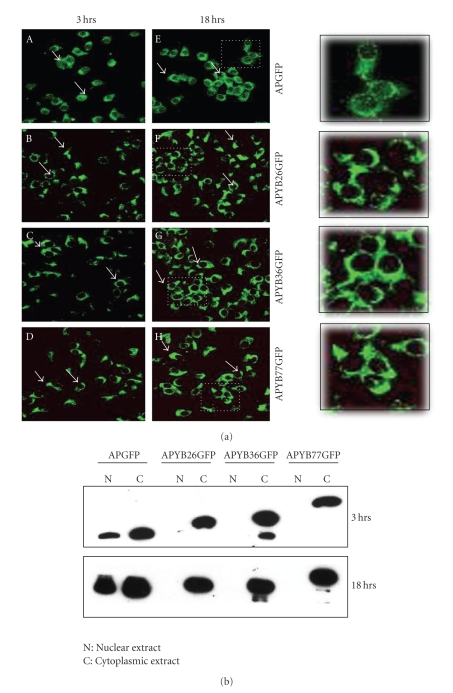
Fusion proteins containing N-terminal YB-1 sequences are restricted to cytoplasm of the cells. (a) Subcellular localization of the internalized proteins in cells incubated with APYB26GFP (panels B and F), APYB36GFP (Panels C and G), and APYB77GFP (Panels D and H) or APGFP control (A and E) are shown. Representative cells are marked by arrows. In order to show in more detail a representative field (marked by a dashed square) from the 18 hour panels was magnified and shown in a separate box on the right of the figure. (b) Immunoblot analysis of nuclear and cytoplasmic extracts using polyclonal GFP antibody. Note that the three APYBGFP proteins are seen only in cytoplasmic extracts at both time points whereas APGFP was detected in both nuclear and cytoplasmic extracts.

 In order to show that the antennapedia peptide can direct the cellular uptake of the YB-1 fusion proteins, we incubated rat hepatoma cells with 40 *μ*g/mL of each protein for 3 and 18 hrs. Cellular extracts were prepared and analyzed by western blotting using anti-GFP antibody. Results presented in Figure 2 indicate that that the fusion proteins are internalized as early as 3 hrs (Figure 2(a), lanes marked L) and continued to accumulate for at least 18 hr (Figure 2). At this time more than 80% of cells have taken up the fusion proteins indicating that the antennapedia peptide allows the cells to take up the proteins efficiently (see Figure 3) Similar results were obtained with primary cells isolated from rat aortic smooth muscle (RASM) (data not shown). These results indicate that the fusion proteins are readily internalized by tumor and primary cells.

### 3.2. N-Terminal Domain of YB-1 Is Responsible for Cytoplasmic Localization

Having demonstrated internalization of all the proteins, we then studied the localization of these proteins within cells. Cells were incubated with the fusion proteins for 3 or 18 hr and prepared for confocal microscopy. Results indicated that more than 80% of the cells had taken up the proteins by 18 hrs. APGFP, which did not contain any YB-1 sequences, was distributed throughout the cell, that is, in both nucleus and cytoplasm (Figure 3(a) panels A and E). This is probably due to the fact that GFP alone can distribute all over the cell unless specific targeting signals are included (reviewed in [26]) or that the cell penetrating antennapedia moiety may be responsible for the translocation of GFP through both cytoplasmic and nuclear membranes. In contrast, incubation of the three YB-1 fusion proteins indicated that both at 3 and 18 hours, most of the protein was localized only in the cytoplasm (Figure 3(a), panels B and D, C and E, and F and H). These results suggest that the amino terminus of YB-1 restricted the fusion proteins to the cytoplasm of cells.

 This was further confirmed by immunoblotting analysis. Nuclear and cytoplasmic extracts of rat hepatoma cells were analyzed by immunoblotting analysis using anti-GFP antibodies. These studies indicated that the APYBGFP fusion proteins were detected only in the cytoplasmic extracts of cells even after 18 hr, whereas the APGFP was detected both in nuclear and cytoplasmic extracts (Figure 3(b)). Less than 2 to 3% of the internalized APYBGFP proteins was found in the nuclei where as in the control APGFP as much as 30 to 40% was detected in the nuclei by 18 hours. We conclude that the presence of N-terminal amino acid sequence of YB-1 in the fusion protein is responsible for their restricted localization to the cytoplasm of cells.

### 3.3. Cells Incubated with APYBGFP Proteins Arrest at the G2/M Phase of the Cell Cycle

**Figure 4:**
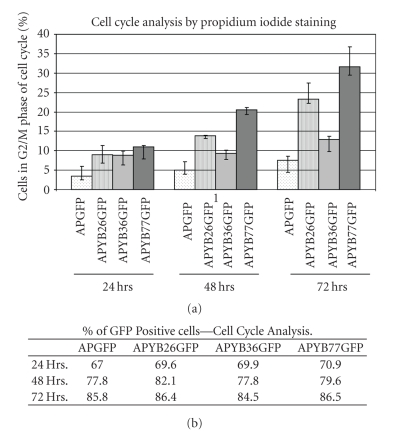
Internalization of YB-1 fusion proteins results in cell cycle arrest at the G2/M phase. Rat hepatoma cells incubated with each of the fusion proteins were stained with propidium iodide and subjected to FACS analysis (a). Three independent experiments were performed for statistical analysis and all the values (per cent of cells) reported are absolute to *P* < .001. Panel B shows the percentage of cells that have taken up the fusion protein as quantified by FACS analysis. This indicates that there is no difference in the extent of protein uptake by cells incubated with the fusion proteins.

**Figure 5:**
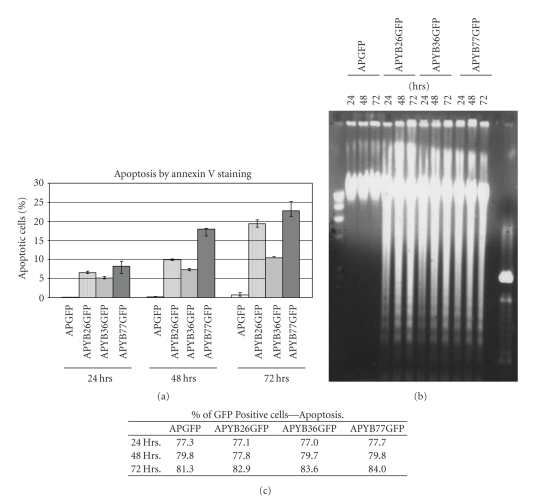
Cells incubated with the three APYBGFP proteins show significant apoptosis. (a) Rat hepatoma cells incubated with each of the fusion proteins were stained with FITC-conjugated annexin V and subjected to FACS analysis. Panel C shows the number of cells (percentage) that have taken up the fusion proteins as quantitated by FACS analysis. (b) Agarose gel electrophoresis of DNA isolated from cells incubated with the fusion. (c) Percentage of cells taken up the fusion protein as quantitated by FACS analysis.

Having demonstrated the delivery of the amino-terminal YB-1 peptides into cells, we wondered whether these YB-1 peptides induce cell cycle defects similar to those we previously observed in YB-1-disrupted heterozygous mutant DT-40 cells [18]. Cells were incubated with each one of these fusion proteins for 24, 48 and 72 hrs. Half of the cells were stained with propidium iodide and subjected to FACS analysis to determine the DNA content. Our results indicate that ~32% of cells were arrested in G2/M phase of the cell cycle following 72 hr incubation with APYB77GFP (Figure 4) while APYB26GFP induced more than 24% of the cells to accumulate in G2/M following the same 72 hr incubation (Figure 4). In contrast, only about 10% APYB36GFP-incubated cells were in G2/M (Figure 5), which is very similar to the APGFP-incubated cells where less than 6%–8% of the cells were in the G2/M phase (Figure 4). These results suggest that the N-terminal sequence of YB-1, containing the alanine-proline-rich domain, may be involved in regulating the progression of cells from G2/M to G1.

 In order to show that the block in G2/M phase was not due to differences in the uptake of the fusion proteins, the remaining half of the samples was used to quantify the mean GFP fluorescence in the GFP positive cells. The results presented in Figure 4(b) indicated that the percentage of GFP-positive cells was similar for all the fusions. In addition, no difference was observed in the mean GFP fluorescence of different fusion proteins. These results suggest that the cell cycle arrest in APYBGFP-incubated cells is not due to variation in the number of fusion protein-positive cells or amount of the internalized proteins, but is due to the presence of YB-1 N-terminal sequence. Further, this data clearly implicate the N-terminus of YB-1 in the arrest of cells at the G2/M phase of the cell cycle.

### 3.4. The Amino Terminus of YB-1 Is Involved in Regulating Apoptosis

Earlier we demonstrated apoptosis in heterozygous mutant DT-40 cells and speculated that this might be due to a dominant negative effect of the potential truncated protein encoded by the defective allele [18]. Subsequently another group reported that down-regulation of YB-1 resulted in an increased rate of apoptotic cell death, demonstrating a direct relationship between YB-1 levels and apoptosis [20]. Based on these observations we hypothesized that the N-terminus of YB-1 is involved in regulating apoptotis. 

 To test the possibility that the N-terminus of YB-1 regulates apoptosis, we carried out experiments that detect the presence of phosphatidylserine on the cell surface using fluorescein-conjugated annexin V and DNA fragmentation, both of which are hallmarks of apoptosis. Cells were incubated with the fusion proteins for 24, 48 and 72 hr and half of the cells were stained with annexin V and analyzed by FACS. Our results indicated that in APGFP-incubated cells less than 1 to 2% of cells were apoptotic after 72 hr incubation (Figure 5(a)). In contrast, incubation with APYB77GFP stimulated 22-23% of cells to become annexin V-positive by 72 hrs. Similar results were obtained with APYB26GFP- incubated cells (Figure 5(a)). By contrast, APYB36GFP is less effective in causing the cells to undergo apoptosis. Again, the uptake of these proteins was comparable as evidenced by the number of GFP positive cells and their mean GFP fluorescence (Figure 5(c)). These results clearly suggest that the N-terminal alanine-proline-rich 26 amino acids is part of a major death domain of YB-1.

 These results were further substantiated by DNA fragmentation analysis. DNA from cells incubated with the fusion proteins for various time periods was analyzed by agarose gel electrophoresis. It can be seen that the pattern of DNA fragmentation, as evidenced by the appearance of the 180–200 bp ladder, was clearly seen with the DNA from cells treated with YB-1 containing fusion proteins (Figure 5(b)) but not with cells incubated with the control fusion protein, APGFP. Futher, staining of the nuclei with DAPI indicated chromatin condensation and broken nuclei in cells treated with the APYBGFP proteins for 72 hr but not in APGFP-incubated cell nuclei which showed normal appearance(see in Supplementary Material Figure S1 available online at doi: 10.155/2009/243532).Based on these results we conclude that the amino terminal region of YB-1 is involved in apoptosis.

### 3.5. Interaction of the N-Terminal Domain of YB-1 with Cyclin D1

**Figure 6:**
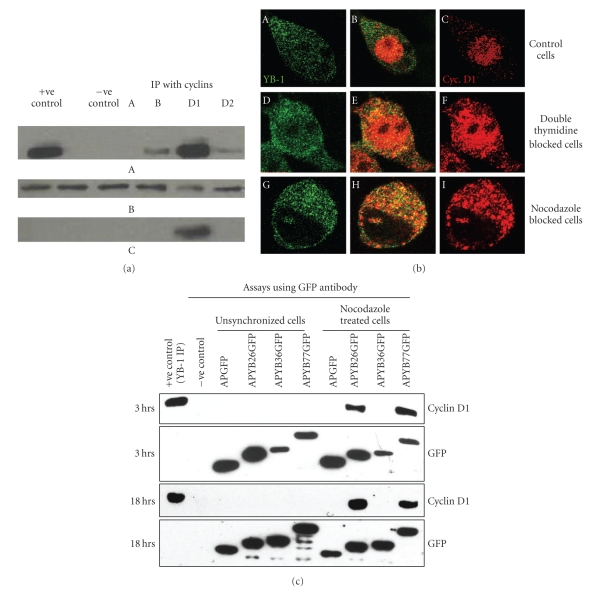
Evidence that YB-1 interacts with Cyclin D1. (a) Immunoprecipitation of rat hepatoma cell extracts with anticyclin antibodies followed by western blotting using YB-1 antibody (top panel marked A). The blot was stripped and reprobed with cyclin D1 antibody (middle panel marked B). Equal amounts of the extracts were also loaded on the gel, blotted and probed with actin (bottom panel marked C). (b) Indirect immunofluorescence analysis of asynchronous and nocodazole-treated cells. YB-1 antibody (FITC-conjugated) or anticyclin D1 antibody (Texas Red) were used to localize YB-1 and cyclin D1 in asynchronous (panels A to C), double thymidine blocked (panels D to E) or nocodazole-treated (G to I) cells. (c) Immunoprecipitation of fusion proteins isolated from whole cells lysates of asynchronous and G2/M phase synchronized cells.

It has been shown that YB-1 regulates transcription of G2/M phase cyclins A and B1 [27]. Since cyclin D1 is upregulated during G2 phase of cell cycle by Ras-mediated translational control of its mRNA, and since it is localization within the nucleus is very important for cell cycle progression, we wondered whether YB-1 interacts with any of these cell cycle-specific cyclins. In order to study this interaction, whole cell extracts of rat hepatoma cells were immunoprecipitated with antibodies for cyclins A, B1, D1 and D2 and examined for the presence of YB-1. Our results clearly indicate that YB-1 predominantly interacts with cyclin D1 as this is the major band observed in the pull-down assays (Figure 6(a)). We observed a minor band with cyclins B and D2 which are also upregulated in G2. In order to show that this interaction is not due to an artifact of protein extraction methods used, we carried out immunoprecipitations of extracts isolated from asynchronous as well as from nocodazole-treated cultures. Nocodazole, as expected, blocked cells in the G2/M (Supplementary Figure S2). Since anticyclin D1 antibody pulled-down YB-1 from nocodazole-treated cell extracts, it indicates that YB-1 interacts with cyclin D1. Reciprocal experiments using anti-YB-1 antibody confirmed that YB-1 indeed binds to cyclin D1 (Supplementary Figure S3). 

 In order to investigate if YB-1 and cyclin D1 co-localize in cells in different phases of the cell cycle, we analyzed their distribution in G1/S and G2/M phase synchronized cells using confocal microscopy. We observed that in the asynchronized and G1/S phase synchronized cells YB-1 was predominantly cytoplasmic (Figure 6(b), Panels A and D) whereas Cyclin D1 was predominantly nuclear (Figure 6(b), Panels C and F) with minimal co-localization (Figure 6(b), Panels B and E). However, in the G2/M phase-blocked cells YB-1 and cyclin D1 were partially co-localized mainly in the cytoplasm (Figure 6(b), Panels G, H and I). 

 In order to show that this colocalization is due to specific interaction between YB-1 and cyclin D1, we carried out immunoprecipitations with isolated nuclear and cytoplasmic extracts using specific antisera (Supplementary Figures S3A and S3B). Anticyclin D1 antibody pulled down only cyclin D1 and anti-YB-1 antibody brought down only YB-1 from the cytoplasm indicating that these proteins are indeed localized in the nuclei and cytoplasm, respectively, of untreated cells. However, in the nocodazole-treated cells, which are arrested in G2 phase, cyclin D1 antibody pulled down YB-1 from cytoplasm and vice versa. As expected, nocodazole blocked a large proportion of cells in G2/M phase of cell cycle (Supplementary Figure S2). We conclude that YB-1 and cyclin D1 interact with each other in the cytoplasm of G2 phase cells. Interestingly when Wortmannin, an inhibitor of PI-3 kinase and Akt pathway was used, a significant amount of cyclin D1 could be detected in the nuclei (Supplementary Figure S4). This indicates that the interaction between YB-1 and cyclin D1 depends on phosphorylation of one or both proteins. Further experiments are necessary to identify the phosporylation sites.

### 3.6. Interaction of the YB-1 N-Terminal Domain with Cyclin D1 in G2/M Phase Blocked Cells

In order to determine if the amino terminus of YB-1 interacts with cyclin D1, whole cell extracts of asynchronized and cells synchronized in G2/M phase using nocodozole were incubated with GFP antibody and probed for cyclin D1. Anti-YB-1 immunoprecipitated Cyclin D1 was used as a positive control. Immunoprecipitation of APGFP incubated cells acted as a negative control. Cyclin D1 immunoprecipitation was not observed in asynchronized cells (Figure 6(c)). However, in nocodazole-blocked cells, cyclin D1 co-precipitated with both APYB26GFP and APYB77GFP at the 3 hr and 18 hr time points (Figure 6(c)), while it was absent in the immunoprecipitates prepared from APYB36GFP-incubated cells. Since asynchronous cultures also contain some cells in G2/M we expect some interaction between cyclin D1 and YB-1, as shown in supplementary Figure S3B. We have not observed such an interaction with the fusion proteins in aynchronous cells. This may be due to sensitivity of the technique or that the interaction in the presence of endogenous YB-1 may be masking detection. Nevertheless, these results suggest that the alanine-proline-rich N-terminal sequence of YB-1 may be involved in sequestering cyclin D1 in the cytoplasm when the cells are blocked in the G2/M phase of cell cycle. Retention of cyclin D1 in the cytoplasm of G2/M phase cells by the YB-1 fusion proteins may be responsible for the observed effects of these fusions on cell cycle progression and apoptosis.

## 4. Discussion

YB-1 expression is closely associated with cell proliferation. For example, YB-1 is highly expressed in regenerating liver and liver cancers but barely detectable in normal adult liver where cells are quiescent [28, 29]. Our earlier studies showed that disruption of one allele of Chk-YB-1b gene resulted in abnormalities in the heterozygous DT-40 mutants, including slower growth rate and increased genomic DNA content that is associated with apoptosis [28]. We speculated that the defects seen may be due to a dominant negative effect of the putative N-terminal truncated protein encoded by the disrupted YB-1 allele. If our assumption was correct, then introduction of peptides corresponding to the amino terminus of YB-1 should mimic the defects that were observed in the mutant DT40 cells [18].

 The antennapedia peptide was shown to be the most efficient and least cytotoxic of all cell penetrating peptides [30]. Therefore the YB-1 sequences were cloned in such a way that the fusion proteins carried the antennapedia peptide at the amino end and a reporter GFP sequence at the carboxyl end. We report here that internalization of the four APGFP fusion proteins occurs at high efficiency in exponentially growing rat hepatoma cells. The control protein, APGFP, which does not have any YB-1 sequence, is distributed throughout the cell—both nuclei and cytoplasm. In contrast, YB-1 fusion proteins are restricted to the cytoplasm of cells. Since APGFP is distributed all over the cell, it appears that the YB-1 moiety is responsible for the cytoplasmic localization. 

 The ability of the YB-1 N-terminus to associate with cyclin D1 and potentially retain it in the cytoplasm of cells may account for the effect of the YB-1 fusions on cell cycle progression and apoptsis. Cyclin D1, encoded by the Bcl-1 gene, is a putative protooncogene, and previous studies have shown that both YB-1 and cyclin D1 are upregulated in many neoplasms [1, 31, 32]. Although YB-1 is generally a cytoplasmic protein and cyclin D1 a nuclear protein, YB-1 is found in the nucleus in mid G1 to S phase [27] at the same time as cyclin D1. Cyclin D1 relocates to the cytoplasm at the end of S phase and into the G2 phase [33, 34], when YB-1 is also concentrated in the cytoplasm. The levels of cyclin D1 in the G2/M phase of cell cycle are crucial for the cells to decide if they have to undergo another round of replication. 

 It has been shown that cyclin D1-knockout mouse embryos survive for about 13 days and then die [35]. Cells isolated from these mice grow normally although at a reduced rate. However, in epithelial cells cyclin D2 compensates for the loss of cyclin D1 indicating the importance of these cyclins in cell proliferation [36]. Cyclin D1 is considered to be a G1 cyclin, as its interaction with Cdk4/6 contributes to the phosphorylation of Rb protein and also sequesters the Cdk inhibitors, which is required for the cell cycle progression to the S phase [37]. 

 Cyclin D1 levels also increase in G2 and are maintained throughout mitosis and G1 [10, 34, 38]. The regulation appears to be at posttranscriptional level, indicating that de novo synthesis of cyclin D1 occurs during G2 phase. Its fate is determined through receptor-coupled Ras signaling [34, 37]. It is likely that when cyclin D1 is present in cytoplasm in G2 that the amino end domain of YB-1 sequesters cyclin D1 and prevents its import into nucleus. Nuclear translocation of YB-1 requires phosphorylation by the serine-threonine protein kinase Akt at ser-102 in the CSD of YB-1 [39–41]. This phosphorylation also induces translation of several cellular mRNAs [42]. Since both the CSD and the carboxy terminal acidic-basic domains are missing in our fusion proteins, many of the functions carried out by the full length endogenous YB-1 molecule are disrupted. As a consequence, the cellular protein(s) that interact with the N-terminal YB-1 sequence affect downstream signaling leading to cellular damage, which keeps the cells in G2, giving an opportunity for cells to recover or undergo apoptosis probably in a p53-dependent pathway [24, 37]. The data provided here suggests that the interaction between YB-1 and cyclin D1 play a critical role in regulating cyclin D1 function at the G2/M phase of the cell cycle. 

 Stabilization and nuclear accumulation of cyclin D1 depends on PI3-K, Akt pathway, which can negatively regulate GSK-3*β* [43], a serine/threonine protein kinase, regulates cyclin D1 expression by regulating mRNA transcription and protein degradation (reviewed in [43]). Phosphorylation of cyclin D1 as well as YB-1 depends on the PI3-K and Akt pathway. YB-1, upon phosphorylation is translocated into nucleus where it directly induces the p110*α* catalytic subunit of PI3-kinase [39, 44]. Similarly, cyclin D1 is also regulated by PI3-kinase, Akt and GSK-3*β* (reviewed in [32]). Cyclin D1 in the nucleus is important for cell cycle progression and export from cytoplasm to nucleus is critical for G2/M esit [34]. Normally cyclin D1 is exported to the cytoplasm where it is degraded by the ubiquitin pathway [32].

 The fact that Wortmannin, an inhibitor of PI3-kinase-Akt pathway, stimulates the nuclear localization of cyclin D1 in nocodazole-treated cells, suggests that the interaction between cyclin D1 and YB-1 and resulting cytoplasmic localization of cyclin D1 depends on the phosphorylation of YB-1 or cyclin D1 or both. YB-1 is known to be phosphorylated on its ser 102 in CSD by both Akt and the p90 ribosomal S6 kinase. This phosphorylation allows YB-1 to shuttle into nucleus where it induces transcriptioin of PI3-K *α*110 catalytic subunit [39]. However, in the APYB77GFP and APYB26GFP, this phosphorylation site is not present and as a result this N-terminal domain sequesters the cyclin D1 and prevents its movement to the nucleus. This could cause the cell cycle to arrest in G2/M. 

 YB-1 is known to interact with several cellular proteins including those involved in DNA synthesis, DNA repair, transcription, and translation. It also binds to several mRNAs and represses translation [42]. Depending on the type of cell and the cell cycle stage these interactions may be required for nuclear translocation and/or for cytoplasmic retention of various cellular factors. It is generally known that cells halted in G2/M are unstable, and during this time the cell survival depends on several proteins including p53, p21 and cyclins, which determine the fate of the cells, that is, whether to go for another round of cell cycle or apoptosis. When full length YB-1 binds to appropriate targets and elicit proper response, the cell cycle progresses normally; however, when the N-terminal sequence binds to proteins such as p53 [45] or cyclin D1 and keeps them in a different compartment of the cell, then it cannot elicit the same response and hence the defects. 

 It has been shown that cells blocked in the G2/M phase of cell cycle appear to be unstable and undergo apoptosis [33, 34, 46]. In addition, it is known that repair of any damaged DNA takes place during G2 and YB-1 has been implicated in this process. In our experiments we found that the N-termainal region of YB-1 stimulated cells to arrest in the G2/M phase of the cell cycle. This dominant effect of the N-terminus of YB-1 may be due to the ability of YB-1 to regulate centrosome function by binding to actin [47] and centrosomes during mitosis [48]. Inhibition of centrosome function can lead to chromosomal instability and aneuploidy [46, 49], which may lead to a block in cell cycle and apoptosis. Our data, which are consistent with these previous observations, suggest a critical role for the YB-1 N-terminal domain in regulating cell proliferation and apoptosis. 

 In summary, in this report we demonstrated co-localization of YB-1 and Cyclin D1 in the cytoplasm of G2/M phase blocked cells. Furthermore, we present evidence for the interaction of Cyclin D1 with fusion proteins APYB26GFP and APYB77GFP in G2/M phase blocked cells. We propose that YB-1 binds to cyclin D1 and sequesters it in the cytoplasm. This causes the cells to arrest in G2/M phase of the cell cycle and protects cyclin D1 from degradation, which is important for G2/M exit. As a consequence of this abnormal arrest in G2/M arrest, the cells become unstable and undergo apoptosis. Since cyclin D1 immunoprecipitates APYB77GFP and APYB26GFP, but not APYB36GFP and correlates with increased apoptosis, it is likely that the interaction with cyclin D1 occurs through the alanine and proline-rich sequence at the N-terminus of YB-1. Further studies are necessary to elucidate the physiological importance of the YB-1/cyclin D1 interaction and to precisely identify the domains involved in this interaction.
